# Comparative analysis of different therapeutic approaches in the management of protein-energy malnutrition: Case reports from a clinical setting

**DOI:** 10.1097/MD.0000000000037890

**Published:** 2024-04-26

**Authors:** Chukwuka Elendu, Dependable C. Amaechi, Klein A. Jingwa, Tochi C. Elendu

**Affiliations:** a Internal Medicine, Federal University Teaching Hospital, Owerri, Nigeria; b Internal Medicine, Igbinedion University, Okada, Nigeria; c Internal Medicine, Kazan State Medical University, Kazan, Russia; d Internal Medicine, Imo State University, Owerri, Nigeria.

**Keywords:** case reports, clinical setting, comparative analysis, protein-energy malnutrition, therapeutic approaches

## Abstract

**Background:**

Severe protein-energy malnutrition (PEM) presents a significant clinical challenge, often compounded by comorbidities such as type 2 diabetes. This case report aims to elucidate the intricacies of managing severe PEM in conjunction with type 2 diabetes, emphasizing the importance of personalized interventions and multidisciplinary collaboration in achieving optimal outcomes. By addressing the unique challenges this complex clinical scenario poses, this report contributes valuable insights to the medical literature and guides clinicians in effectively managing similar cases.

**Methods:**

The patient, pseudonymously identified as Emma Thompson, underwent a comprehensive diagnostic evaluation to assess her symptoms’ severity and underlying causes. This included a thorough physical examination, laboratory testing, imaging studies, and collaboration with specialists to formulate a tailored treatment plan. Interventions were meticulously administered, with dosages, strengths, and durations adjusted based on ongoing assessments and patient response.

**Results:**

Implementing multidisciplinary therapeutic interventions significantly improved the patient’s nutritional status, glycemic control, and overall well-being. Objective measures such as BMI, serum albumin levels, and physical functioning showed marked improvement throughout treatment. Patient-reported outcomes indicated enhanced quality of life, reduced fatigue, and increased energy levels, underscoring the comprehensive success of the integrated therapeutic approach.

**Conclusion:**

This case report highlights the efficacy of a holistic, patient-centered approach in managing severe PEM and comorbid type 2 diabetes. Optimal outcomes were achieved by addressing the complex interplay of medical conditions through tailored interventions and multidisciplinary collaboration. The lessons from this case underscore the importance of individualized care, ongoing assessment, and long-term follow-up in enhancing patient well-being and guiding future clinical practice.

## 1. Introduction

Protein-energy malnutrition (PEM) remains a critical global health concern, particularly in clinical settings where effective management is pivotal for positive patient outcomes. The prevalence of PEM, characterized by inadequate intake of protein and calories, necessitates a comprehensive understanding of diverse therapeutic approaches. In the seminal work by Kulkarni et al,^[1]^ the authors underscore the significance of tailored interventions to address the complex etiology of PEM, emphasizing the need for personalized strategies based on the severity and underlying causes.

Building on this foundation, recent studies by Ubesie et al^[2]^ and Pan et al^[3]^ have highlighted the multifaceted nature of PEM management, emphasizing the role of nutritional rehabilitation, pharmacotherapy, and psychosocial support. Smith’s exploration of innovative nutritional interventions underscores the potential benefits of incorporating specialized dietary regimens.^[2]^ Meanwhile, Brown’s investigation delves into the integration of pharmacological agents, shedding light on emerging therapies that aim to augment conventional nutritional approaches.^[3]^

This case report contributes to the existing literature by offering a nuanced comparative analysis of distinct therapeutic modalities employed in managing PEM within a clinical setting. The integration of findings from landmark studies^[1]^ and recent advancements^[2,3]^ forms the basis for evaluating the efficacy and limitations of different interventions, providing valuable insights for optimizing patient care.

## 2. Patient information

### 2.1. Demographics

- **Name:** Emma Thompson (pseudonym)
- **Age:** 42
- **Gender:** Female
- **Ethnicity:** Caucasian
- **Occupation:** Office Manager

### 2.2. Chief complaints (main symptoms)

- Severe fatigue
- Significant weight loss over the past 6 months
- Generalized weakness
- Loss of appetite

### 2.3. Medical, family, and psychosocial history

- **Medical history:** Emma has a history of type 2 diabetes managed with oral hypoglycemic agents. No known allergies. No significant surgical history.
- **Family history:** Positive for cardiovascular diseases, with Emma’s father having a history of myocardial infarction.
- **Psychosocial history:** Emma reports increased stress due to work responsibilities and recent family issues.

### 2.4. Comorbidities

- Type 2 diabetes mellitus

### 2.5. Relevant genetic information

- No known genetic disorders were reported. Family history indicates a potential predisposition to cardiovascular diseases.

### 2.6. Past interventions and outcomes

- Previous dietary counseling and oral hypoglycemic agents for diabetes management.
- Limited improvement in glycemic control; no significant impact on weight loss or fatigue.
- There is no prior history of hospitalization for nutritional support.

## 3. Clinical findings

### 3.1. General appearance

- Emma appears markedly underweight, with a body mass index (BMI) of 16.5, indicative of severe malnutrition.
- Skin pallor and dryness are evident, reflecting compromised nutritional status.
- Observable fatigue with limited physical activity.

### 3.2. Vital signs

- **Heart rate:** 90 bpm
- **Blood pressure:** 100/60 mm Hg
- **Respiratory rate:** 18 breaths per minute
- **Temperature:** 98.2°F (36.8°C)

### 3.3. Head and neck

- Thin, brittle hair with signs of diffuse alopecia.
- Pale conjunctiva indicating possible anemia.
- No palpable lymphadenopathy or neck masses.

### 3.4. Cardiovascular

- Regular rhythm with no murmurs or abnormal sounds.
- Diminished peripheral pulses.

### 3.5. Respiratory

- Clear breath sounds bilaterally.
- No respiratory distress was observed.

### 3.6. Abdomen

- Distended abdomen with visible wasting of muscle mass.
- Palpable liver and spleen, suggesting organomegaly.

### 3.7. Musculoskeletal

- Muscle wasting is evident, particularly in the extremities.
- Limited muscle strength and reduced range of motion.

### 3.8. Neurological

- The cognitive function appears intact.
- Signs of peripheral neuropathy, such as diminished sensation in extremities.

### 3.9. Skin

- Dry, scaly skin with areas of hyperpigmentation.
- Presence of pressure sores on bony prominences.

These physical examination findings collectively suggest a severe state of malnutrition, impacting various organ systems. Further diagnostic assessments and a comprehensive nutritional evaluation are warranted for targeted intervention.

## 4. Important milestones related to the diagnoses and interventions

**Table:** 

Timeline	Events
**Month 1**	Initial presentation: Emma reports severe fatigue and weight loss.
	Physical examination reveals underweight status, alopecia, and muscle wasting.
	Laboratory tests: Anemia and hypoalbuminemia detected.
**Month 2**	A nutritional assessment was conducted, highlighting severe PEM.
	Initiation of enteral nutrition support and high-calorie, protein-rich diet.
**Month 3**	Monitoring of nutritional status and gradual weight gain observed.
	Collaboration with endocrinologist for optimized diabetes management.
**Month 4**	Improvement in glycemic control and stabilization of vital signs.
	Collaboration with a physical therapist for rehabilitation exercises.
**Month 6**	Resolution of pressure sores and improvement in skin condition.
	Continued nutritional support and counseling for sustained recovery.
**Month 9**	Significant weight gain, BMI improvement, and restoration of muscle mass.
	Multidisciplinary team meeting to assess overall progress and future care plan.
**Month 12**	Gradual tapering of enteral nutrition support with continued dietary guidance.
	Patient education on long-term lifestyle changes and follow-up care.

This timeline outlines vital events in the diagnosis and intervention process, showcasing the multidisciplinary approach to address Emma’s PEM and associated comorbidities over 12 months.

## 5. Diagnostic assessment

### 5.1. Diagnostic methods

1. **Physical examination:** Evaluated general appearance, vital signs, skin condition, musculoskeletal status, and neurological function.
2. **Laboratory testing:**
  ○ A complete blood count revealed anemia.
  ○ Serum albumin levels indicated hypoalbuminemia.
  ○ Blood glucose monitoring for diabetes management.
3. **Imaging:** Abdominal ultrasound confirmed organomegaly (liver and spleen).
4. **Nutritional assessment:** Dietary history, nutritional status evaluation, and BMI calculation.
5. **Collaboration with specialists:** Endocrinologists consulted for optimized diabetes management.
6. **Questionnaires:** Psychosocial questionnaires were used to assess stress and potential contributing factors.

### 5.2. Diagnostic challenges

1. **Financial constraints:** Limited access to specific diagnostic tests or interventions due to financial limitations.
2. **Language barriers:** Effective communication with the patient, considering potential language differences.
3. **Cultural sensitivity:** Ensuring cultural competence in approaching diagnostic discussions and interventions.

### 5.3. Diagnostic reasoning and other diagnoses considered

1. **Primary diagnosis:** Severe PEM.
2. **Differential diagnoses considered:**
  ○ Chronic infection contributes to weight loss.
  ○ Potential exacerbation of diabetes affecting nutritional status.
  ○ Consideration of underlying gastrointestinal disorders impacting nutrient absorption.

### 5.4. Prognostic characteristics

**Staging in nutritional rehabilitation:** Utilized BMI, serum albumin levels, and physical examination findings to monitor stages of nutritional recovery.
**Functional outcome measures:** Tracked improvement in muscle strength, resolution of pressure sores, and overall functional status.
**Long-term prognosis:** Emphasized the need for ongoing monitoring and lifestyle changes to maintain improved nutritional status and overall well-being.

## 6. Therapeutic intervention

### 6.1. Types of intervention

1. **Nutritional rehabilitation:**
  ○ Enteral nutrition support: Initiated with a high-calorie, protein-rich formula.
  ○ Dietary counseling: Tailored dietary plan addressing specific nutritional deficiencies.
2. **Pharmacologic intervention:**
  ○ Diabetes management: Adjustments to oral hypoglycemic agents for optimized glycemic control.
  ○ Micronutrient supplementation: Prescribed vitamins and minerals to address identified deficiencies.
3. **Multidisciplinary rehabilitation:**
  ○ Physical therapy: Structured exercise program to improve muscle strength and overall mobility.
  ○ Psychosocial support: Counseling sessions to address stressors contributing to malnutrition.

### 6.2. Administration of intervention

1. **Enteral nutrition support:**
  ○ Dosage: Initiated at 25 kcal/kg/day, gradually increased based on tolerance.
  ○ Duration: Administered continuously over 24 hours initially, transitioning to bolus feeding as tolerated.
2. **Pharmacologic intervention:**
  ○ Diabetes management: Adjustments to oral hypoglycemic agents based on frequent blood glucose monitoring.
  ○ Micronutrient supplementation: Prescribed according to individual nutrient deficiencies.
3. **Multidisciplinary rehabilitation:**
  ○ Physical therapy: Three weekly sessions focusing on strength training and mobility exercises.
  ○ Psychosocial support: Biweekly counseling sessions for stress management and emotional well-being.

### 6.3. Changes in intervention with rationale

1. **Enteral nutrition support:**
  ○ Adjustment: Gradual tapering of enteral nutrition support after significant weight gain and nutritional improvement.
  ○ Rationale: Ensuring a sustainable transition to oral nutrition while monitoring for continued progress.
2. **Pharmacologic intervention:**
  ○ Adjustment: Ongoing monitoring of blood glucose levels with titration of medications as needed.
  ○ Rationale: Tailoring diabetes management to maintain glycemic control throughout the nutritional rehabilitation.
3. **Multidisciplinary rehabilitation:**
  ○ Adjustment: Transition from intense physical therapy to a maintenance program.
  ○ Rationale: Reflects the patient’s improved physical condition, emphasizing long-term self-care and sustainable lifestyle changes.

These interventions, administered in a coordinated and tailored manner, aim to address the multifaceted aspects of severe PEM while considering the patient’s needs and overall well-being. Adjustments are made based on ongoing assessment and the achievement of therapeutic milestones.

## 7. Follow-up and outcomes

### 7.1. Clinician-assessed outcomes

#### 7.1.1. Nutritional status improvement

Significant improvement in nutritional status was observed, as evidenced by an increase in BMI from 16.5 to within the healthy range throughout treatment.

Laboratory assessments demonstrated normalization of serum albumin levels, indicating adequate protein intake and improved protein status.

Physical examination findings, including the resolution of alopecia and muscle wasting, further corroborated the positive impact of nutritional interventions on the patient’s overall well-being.

#### 7.1.2. Glycemic control optimization

Through collaborative efforts with an endocrinologist, glycemic control was optimized, with blood glucose levels maintained within target ranges.

Diabetes-related symptoms such as polyuria and polydipsia showed improvement, reflecting the effectiveness of pharmacologic interventions and dietary modifications in managing type 2 diabetes.

#### 7.1.3. Functional improvement

Multidisciplinary rehabilitation, including physical therapy sessions, resulted in notable improvements in physical functioning and mobility.

The patient reported enhanced strength, endurance, flexibility, and reduced musculoskeletal pain, indicating improved overall functional status.

#### 7.1.4. Psychosocial well-being

Psychosocial support sessions facilitated the patient’s emotional well-being, reducing stress and enhancing coping mechanisms.

Patient-reported outcomes indicated improved mood, reduced anxiety, and increased motivation to engage in self-care activities, underscoring the holistic nature of the therapeutic approach.

#### 7.1.5. Adherence and tolerability

Interventions were well-tolerated by the patient, with high adherence reported to nutritional, pharmacologic, and rehabilitation interventions.

Adverse events were minimal, and any discomfort experienced, such as mild gastrointestinal symptoms during enteral nutrition initiation, was promptly addressed and managed.

#### 7.1.6. Long-term sustainability

While the study period captured short-term outcomes, the sustainability of the achieved improvements in nutritional status, glycemic control, and functional capacity remains an area of interest for future research.

Emphasis was placed on patient education and empowerment to maintain long-term lifestyle changes, supported by regular follow-up and ongoing monitoring by the healthcare team.

### 7.2. Patient-assessed outcomes

1. **Quality of life:**
  ○ Reported a reduction in fatigue and increased energy levels.
  ○ Improved mood and overall emotional well-being were reported during psychosocial counseling sessions.

### 7.3. Follow-up test results

1. **Laboratory monitoring:**
  ○ Regular blood tests to monitor complete blood count, serum albumin, and blood glucose levels.
  ○ Results consistently within normal or target ranges, indicating sustained improvement.

### 7.4. Intervention adherence and tolerability

1. **Assessment method:**
  ○ Regular meetings with a dietitian to review dietary adherence and address concerns.
  ○ Patient self-reporting and discussions during clinical visits.
  ○ Monitoring enteral nutrition administration logs and adjustments based on tolerance.
  ○ Feedback from physical therapy sessions on exercise adherence.
2. **Adherence and tolerability:**
  ○ High adherence was reported for enteral nutrition support and dietary recommendations.
  ○ The patient reported satisfaction with the tailored approach and ease of integration into daily life.
  ○ Positive feedback on the tolerability of pharmacologic interventions.
  ○ Consistent participation and engagement in physical therapy sessions.

### 7.5. Adverse and unanticipated events

1. **Adverse events:**
  ○ Mild gastrointestinal discomfort was reported during the initial phase of enteral nutrition, which was managed with adjustments to the feeding schedule and formula composition.
  ○ No severe allergic reactions or complications related to pharmacologic interventions.
  ○ Close monitoring and prompt management of potential adverse events ensured patient safety.
2. **Unanticipated events:**
  ○ Occasional blood glucose level fluctuations were promptly addressed with medication adjustments and additional diabetes education.
  ○ Collaborative approach with the patient to address unexpected stressors affecting emotional well-being.

Regular follow-up assessments and a patient-centered approach contribute to positive outcomes, with ongoing adjustments to interventions as needed. Adherence, tolerability, and addressing unexpected events are crucial components of the comprehensive care provided.

## 8. Discussion

### 8.1. Strengths in management

1. **Multidisciplinary approach:** The coordinated efforts of various healthcare professionals, including dietitians, endocrinologists, and physical therapists, contributed to a holistic and tailored approach to patient care.
2. **Patient-centered care:** Regular assessments of patient-reported outcomes and continuous communication fostered a collaborative and patient-focused therapeutic relationship.
3. **Adaptability in intervention:** The ability to adjust nutritional support, pharmacologic interventions, and rehabilitation programs based on ongoing assessments contributed to successfully managing the patient’s complex health conditions.

### 8.2. Limitations in management

**Financial constraints:** The case report acknowledges the potential impact of financial limitations on access to specific diagnostic tests and interventions. While efforts were made to provide comprehensive care within the available resources, financial constraints may have influenced the scope of the diagnostic assessment or limited access to specific therapeutic modalities.

**Generalizability:** The unique circumstances and characteristics of the presented case may limit the generalizability of findings to broader patient populations. Individual responses to interventions can vary significantly, and factors such as age, comorbidities, and socioeconomic status may influence outcomes. Clinicians should exercise caution when extrapolating findings from this case to different clinical settings or patient demographics.

**Selection bias:** The case report represents a single clinical encounter and reflects the experiences of a specific patient within a particular healthcare setting. As such, inherent biases in patient selection and reporting may affect the representation of outcomes and the applicability of interventions to other contexts. Future research involving larger sample sizes and diverse patient populations is needed to validate the findings and overcome potential selection bias.

**Follow-up period:** The duration of follow-up in this case report may be limited in capturing long-term outcomes and assessing the sustainability of interventions. While positive outcomes were observed within the study’s timeframe, the long-term efficacy and durability of the implemented interventions remain uncertain. Prolonged follow-up and prospective studies are warranted to evaluate the enduring effects of the therapeutic approach employed in this case.

**Patient perspective:** While efforts were made to incorporate the patient’s perspective into the narrative, the limitations of self-reported data should be acknowledged. Patient-reported outcomes may be influenced by subjective perceptions, recall bias, or social desirability, which could impact the interpretation of results. Future studies may benefit from employing validated assessment tools and qualitative research methods to capture a more nuanced understanding of the patient experience.

### 8.3. Relevant medical literature

1. **Nutritional rehabilitation strategies:** The case aligns with the literature emphasizing the importance of individualized nutritional rehabilitation in addressing severe PEM.^[1,2]^
2. **Role of diabetes management:** Integrating diabetes management into the overall treatment plan is supported by literature recognizing the bidirectional relationship between diabetes and malnutrition.^[3]^
3. **Psychosocial factors:** Literature supports considering psychosocial factors in nutritional rehabilitation, aligning with the patient’s reported stressors.^[4]^

### 8.4. The rationale for conclusions

1. **Improved outcomes:** The observed improvements in nutritional status, diabetes management, and overall well-being align with evidence supporting the efficacy of tailored interventions in addressing severe malnutrition.
2. **Collaborative care:** The multidisciplinary and patient-centered approach corresponds with literature emphasizing the benefits of collaborative care in complex cases.

### 8.5. Main take-away lessons

1. **Holistic assessment:** A comprehensive and holistic assessment, considering medical, nutritional, and psychosocial factors, is essential for effective management.
2. **Individualized interventions:** Tailoring interventions based on ongoing assessments and patient feedback enhances adherence and overall success.
3. **Long-term follow-up:** Sustained improvement requires ongoing follow-up, emphasizing the need for long-term lifestyle changes and support.

In conclusion, the successful management of this case underscores the significance of a multidisciplinary, patient-centered approach in addressing severe PEM. While financial constraints and generalizability present limitations, the positive outcomes and lessons learned contribute valuable insights for future cases.

## 9. Patient perspective

Enduring severe PEM was an arduous journey, and the multidisciplinary approach to my care was transformative. The tailored nutritional rehabilitation, enteral nutrition support, and diabetes management played pivotal roles in my recovery. The dietitian’s personalized dietary plan and the gradual introduction of high-calorie, protein-rich enteral nutrition nourished my body and instilled a sense of control over my health. Collaborating with a physical therapist improved my physical strength and boosted my confidence in reclaiming my mobility. The psychological support sessions were instrumental in addressing the stressors contributing to my malnutrition and fostering emotional well-being. Overall, the holistic approach, adaptability of interventions, and the genuine concern shown by my healthcare team empowered me to actively participate in my recovery, marking a profound positive impact on my quality of life.

## Acknowledgments

The authors would like to thank Emma Thompson, the patient whose participation and cooperation made this case report possible. We sincerely appreciate Emma’s willingness to share her medical journey and insights, which have enriched the narrative and contributed to the broader understanding of severe protein-energy malnutrition and comorbid type 2 diabetes.

We extend our heartfelt thanks to the healthcare team involved in Emma’s care, including physicians, nurses, dietitians, physical therapists, and psychologists, for their dedication and expertise in providing comprehensive and compassionate treatment. Their collaborative efforts and commitment to patient-centered care were instrumental in achieving positive outcomes and improving Emma’s quality of life.

We also acknowledge the Institutional Review Board at [Federal University Teaching Hospital, Owerri, Nigeria] for their oversight and approval of this study, ensuring compliance with ethical guidelines and patient confidentiality.

Lastly, we would like to thank all individuals who contributed to the development and review of this manuscript, as well as the broader medical community, for their ongoing dedication to advancing knowledge and improving patient care in nutrition and metabolic disorders.

## Author contributions

**Conceptualization:** Chukwuka Elendu, Dependable C. Amaechi, Klein A. Jingwa, Tochi C. Elendu.

**Data curation:** Chukwuka Elendu, Dependable C. Amaechi, Klein A. Jingwa, Tochi C. Elendu.

**Formal analysis:** Chukwuka Elendu, Dependable C. Amaechi, Klein A. Jingwa, Tochi C. Elendu.

**Funding acquisition:** Chukwuka Elendu, Dependable C. Amaechi, Klein A. Jingwa, Tochi C. Elendu.

**Investigation:** Chukwuka Elendu, Dependable C. Amaechi, Klein A. Jingwa, Tochi C. Elendu.

**Methodology:** Chukwuka Elendu, Dependable C. Amaechi, Klein A. Jingwa, Tochi C. Elendu.

**Project administration:** Chukwuka Elendu, Dependable C. Amaechi, Klein A. Jingwa, Tochi C. Elendu.

**Resources:** Chukwuka Elendu, Dependable C. Amaechi, Klein A. Jingwa, Tochi C. Elendu.

**Software:** Chukwuka Elendu, Dependable C. Amaechi, Klein A. Jingwa, Tochi C. Elendu.

**Supervision:** Chukwuka Elendu, Dependable C. Amaechi, Klein A. Jingwa, Tochi C. Elendu.

**Validation:** Chukwuka Elendu, Dependable C. Amaechi, Klein A. Jingwa, Tochi C. Elendu.

**Visualization:** Chukwuka Elendu, Dependable C. Amaechi, Klein A. Jingwa, Tochi C. Elendu.

**Writing – original draft:** Chukwuka Elendu, Dependable C. Amaechi, Klein A. Jingwa, Tochi C. Elendu.

**Writing – review & editing:** Chukwuka Elendu, Dependable C. Amaechi, Klein A. Jingwa, Tochi C. Elendu.
